# Immune modulation of the brain-gut-microbe axis

**DOI:** 10.3389/fmicb.2014.00146

**Published:** 2014-04-07

**Authors:** Sahar El Aidy, Timothy G. Dinan, John F. Cryan

**Affiliations:** ^1^Laboratory of Neurogastroenterology, Alimentary Pharmabiotic Centre, University College CorkCork, Ireland; ^2^Department of Industrial Biotechnology, Genetic Engineering and Biotechnology Research Institute, Sadat City UniversitySadat City, Egypt; ^3^Department of Psychiatry, University College CorkCork, Ireland; ^4^Department of Anatomy and Neuroscience, University College CorkCork, Ireland

**Keywords:** neuropeptides, immune cells, gut microbiota, nervous system, HPA

Only recently have we fully appreciated that the classically separated domains of neurology, endocrinology, immunology and microbiology, with their various organs- the brain, glands, gut, immune cells and microbiota, could actually be joined to each other in a multidirectional network of communication, in order to maintain homeostasis. For example, local and systemic immune activation have profound neural and behavioral effects (Campos-Rodríguez et al., 2013), neuroendocrine hormones regulate immune cytokines, and together, the nervous system and immune system work together in synergy to protect the body from infection (Steinman, 2004). Analogously, the gut microbes greatly impact the host immunological, psychological, and overall well-being of the host (Collins and Bercik, 2013; El Aidy and Kleerebezem, 2013a; Wang and Kasper, 2013; Moloney et al., 2014). However, definitive mechanisms that orchestrate a functionally relevant communication within this network, in particular, during the early life development, are yet to be elucidated. A potential unifying mechanism very likely involves multiple-functioning molecules and their receptors, as they are produced by, act upon and move from one system to another linking the brain, gut, immune system, and microbiota. These messenger molecules include (among others) neurotransmitters, neuropeptides, endocrine hormones, and cytokines.

The physiological phenomenon of maturation of the immune and neurological systems, as well as the microbial colonization, initiated within the fetal period, are dynamic in their character and are expanding in time through the first months and even years of human's life. The mode of delivery, be it vaginal birth or caesarean-section, has recently been shown to be critical in determining the pioneer microbial composition of neonates (Dominguez-Bello et al., 2010). Particularly over the first few years of life, as the microbiota develops, there is a greater potential for disruption of the long-term microbial state upon the repeated use of antibiotics (Lemon et al., 2012). Not only does the antibiotics use disrupt the microbial community in healthy infants but also it amplifies the microbial dysbiosis in pediatric patients with Crohn's disease (Gevers et al., 2014). Several reports have shown that dysbiosis alone as may result from antibiotic treatment is sufficient to drive intestinal inflammation (Hooper et al., 2012). Additionally, alterations of the microbial composition are often associated with changes in brain development and plasticity and alterations in motor, anxiety and social behavior (Sudo et al., 2004; Diaz-Heijtz et al., 2011; Neufeld et al., 2011; Clarke et al., 2013; Desbonnet et al., 2014). Consequently, abrupt shifts during the infant's unique developmental path through this early unstable phase may have longer term health implications (Costello et al., 2012). Activation of the (innate) immune response during the primary colonization involves the induction of toll-like receptors (TLRs) (Carvalho et al., 2012; El Aidy et al., 2012a, 2013b) and is linked to the stress induced by colonization, which increases gut permeability (Dinan and Cryan, 2012) to the colonizing microbes and their metabolites. Although very crucial in the recognition of the gut microbes, the scenario of immune activation via TLRs appear not to respond promptly at the initial stage of colonization (El Aidy et al., 2013c), suggesting that the very rapid actions of neurotransmitters and hormones might be important to control the priming and migration of cells of the first line of defense. Several members of pioneer gut colonizers are able to produce neurotransmitters. *Escherichia* and *Streptococcus*, for instance, can produce norepinephrine and serotonin (5-HT), whereas, *Lactobacillus* and *Bifidobacterium* can produce GABA and acetylcholine (Roshchina, 2010; Lyte, 2011). Mobile cells of the immune system express receptors for neurotransmitters (Pert et al., 1985). For example, migration of immature dendritic cells (DCs) to lymph nodes is mediated via α1 adrenergic receptors (Maestroni, 2000), emphasizing the early effects of the sympathetic nervous system (SNS) at the start of a local immune response. Norepinephrine, a neurotransmitter of the SNS (among other molecules stored in sympathetic vesicles), has pro-inflammatory effects at low concentration mediated through binding to α2 adrenoceptors and reduction of cAMP levels (Spengler et al., 1990). On the other hand, acetylcholine, the principle vagal neurotransmitter, attenuates the release of cytokines [tumor necrosis factor-alpha (TNF- α), interleukin (Il)-1β, Il-6 and Il-18, but not the anti-inflammatory cytokine Il-10, in lipopolysaccharides (LPS) stimulated human macrophage cultures (Borovikova et al., 2000)]. Moreover, choline acetyltransferase (ChAT), the key enzyme in the synthesis of acetylcholine, is expressed by B cells, DCs, and macrophages in the mucosal-associated lymphoid tissue (MALT). Reardon et al. reported that ChAT expression begins after microbial colonization, following birth, and requires MyD88-dependent signaling derived from the intestinal microbiota (Reardon et al., 2013). Monocytes such as macrophages and other leucocytes travel through the blood and when they come within scenting distance of a given neurotransmitter, they begin to chemotaxically orient toward it, and then communicate with other immune cells in the adaptive arm such as B and T lymphocytes to ensure well-coordinated immune response.

In parallel, activation of the nervous system, such as afferent vagus nerve fibers by cytokines stimulates neuronal anti-inflammatory responses (Sternberg, 1997). Immune cells can produce various neurotransmitters and other factors, which alert the brain to the changes that occur in the body and affect the plasticity of the local and central nervous systems, thereby can regulate mood and behavior. Leukocytes, for example, synthesize and release corticotropin (ACTH) and endorphins in response to bacterial LPS (Harbour-McMenamin et al., 1985). Mounting an innate immune response during pathogenesis and transiently, during primary gut colonization results in the production of pro-inflammatory cytokines such as Il-1β, TNF- α, and IFN-γ (El Aidy et al., 2013c). The pro-inflammatory cytokine- Il-1β, for instance, is able to inhibit the release of norepinephine from noradrenoceptor axon terminals, in the intestine via the induction of nitric oxide (Rühl et al., 1994; Rühl and Collins, 1997). To note, norepinephrine via β adrenergic signaling, inhibits many aspects of the innate and Th1 mediated immune response (Straub et al., 2006), whereas activated macrophages and other innate immune cells produce nerve repellent factors directed toward sympathetic nerve to counteract the sympathetic inhibitory effect (Miller et al., 2004). Importantly, another possible mechanism underlying the reduced levels of norepinephrine during mounting innate and Th1 mediated immune responses is the decreased L-DOPA decarboxylase activity-the enzyme that converts L-DOPA to norepinephrine, as observed in both inflamed and non-inflamed colonic mucosa of Crohn's patients (Magro et al., 2002).

One mechanism through which immune activation or immunomodulation may affect physiology and behavior is via actions on serotonergic systems (Lowry et al., 2007). Analogous to the action of gut microbiota during primary colonization, Lowry et al. found that the non-pathogenic *Mycobacterium vaccae* led to stimulation of the peripheral immune system. The Th1 and Treg but not Th2 mediated immune activation stimulated a specific subset of serotonergic neurons in the dorsal raphe nucleus of mice and increased serotonin metabolism within the ventromedial prefrontal cortex (Lowry et al., 2007). This finding demonstrates that the type of peripheral immune response is important in determining the effects on serotonergic neurons. Additionally, the study of Lowry and co-workers suggests that the immune-responsive subpopulation of serotonergic neurons in the dorsal raphe appears to play an important role in the regulation of mood during the health state. Afferent fibers within the vagus nerve could be involved in transferring signals of peripheral immune activation to the CNS (Maier et al., 1998). The regulating mechanism appears to involve enhancement of c-Fos expression in dorsal raphe nucleus serotonergic neurons (Hollis et al., 2006). It is therefore tempting to speculate that immune activation under physiological conditions stimulates a subset of serotonergic neurons, distinct from those activated under pathological conditions or by uncontrollable stressors. Nevertheless it is unclear if this association reflects a causal or reactionary response. Immune activation induces symptoms of depression and anxiety in human patients including disorders such as irritable bowel syndrome (IBS), a disorder of the gut-brain axis (Clarke et al., 2009) and in patients receiving treatment with interferon (Felger et al., 2013). Treatment with serotonergic antidepressant drugs prevents the onset of depressive symptoms in such situations (Capuron and Miller, 2004; Capuron et al., 2004). Moreover, recent data has shown that the TNF antagonist infliximab reduces depression symptoms in a subset of patients with high baseline inflammatory biomarkers (Raison et al., 2013). Of note, local and systemic depletion of the 5-HT precursor- Tryptophan (Trp) is associated with elevation of the immunomodulatory enzyme Indoleamine-pyrrole 2,3-dioxygenase (IDO), which occurs during immune activation (Moffett and Namboodiri, 2003) and transiently during primary gut colonization (El Aidy et al., 2012b, 2013b). Moreover, 5-HT is altered by the gut microbiota, being elevated in conventionally raised mice, only when gut colonization occurs at birth (Clarke et al., 2013). Collectively, these findings illustrate evidence that serotonergic systems may play an important role in the relationship between the immune function, gut microbiota, and psychological state.

Local and systemic elevation of pro-inflammatory cytokines, in particular, Il-1β and Il-6 and altered 5-HT levels cause activation of the hypothalamic-pituitary-adrenal (HPA) axis and production of corticotrophin releasing factor (CRF). CRF, which is also elevated in stress response, causes disturbance to other neuropeptides, leading to changes in mood and behavior (Dinan and Cryan, 2012). CRF stimulates the anterior pituitary gland to release the stress hormone; ACTH, which in turn stimulates release of cortisol from the adrenal gland. Cortisol, through a feedback loop, regulates the levels of CRF, and ACTH. Importantly, immune cells, through the COX-2 pathway and production of PEG-2 during (pro-) inflammation, stimulate the adrenal gland to produce corticosterone, which exclusively supports the β adrenergic pathway through which the SNS perform its anti-inflammatory effect (Straub et al., 2006). This indicates that under normal conditions the neuroendocrine and immune systems coordinate to ensure maintenance of homeostasis. Indeed, patients with IBS show inadequately low concentration of the anti-inflammatory steroid hormone, cortisol (Straub et al., 2002).

In conclusion, we assume that the intimate communication between the microbiota-immune-neuroendocrine systems involves multiple-functioning molecule(s). Those same molecule(s) appear to be produced by and signal member(s) of the gut microbial community, the neuroendocrine and immune systems to mount or avoid rising an attack response against the commensals. This would be of crucial importance early in life. During this vulnerable period, it is believed to exist a narrow window during which colonization with a “healthy” microbiota exerts effects that may decrease susceptibility to diseases and ensure normal development of the mucosal and systemic immunity and metabolism as well as the development of HPA axis,which impacts on the gut through its action on the enteric nervous system, immune system and the CNS (Sudo et al., 2004; Shreiner et al., 2008). The most important question needed to be answered at this point, what would be the molecular mechanisms underlying the intimate cross-talk between the immune system and the microbiota-gut-brain axis at its various nodes of interaction?

## Conflict of interest statement

The authors declare that the research was conducted in the absence of any commercial or financial relationships that could be construed as a potential conflict of interest.
